# Uniting Drug and Delivery: Metal Oxide Hybrid Nanotherapeutics for Skin Wound Care

**DOI:** 10.3390/pharmaceutics12080780

**Published:** 2020-08-17

**Authors:** Martin T. Matter, Sebastian Probst, Severin Läuchli, Inge K. Herrmann

**Affiliations:** 1 Nanoparticle Systems Engineering Laboratory, Institute of Energy and Process Engineering, Department of Mechanical and Process Engineering, ETH Zurich, Sonneggstrasse 3, 8092 Zurich, Switzerland; tino.matter@empa.ch; 2 Laboratory for Particles-Biology Interactions, Department of Materials Meet Life, Swiss Federal Laboratories for Materials Science and Technology (Empa), Lerchenfeldstrasse 5, 9014 St. Gallen, Switzerland; 3 School of Health Sciences, HES-SO University of Applied Sciences and Arts Western Switzerland, Avenue de Champel 47, 1206 Geneva, Switzerland; sebastian.probst@hesge.ch; 4 Department of Dermatology, University Hospital Zurich, Rämistrasse 100, 8091 Zurich, Switzerland; severin.laeuchli@usz.ch

**Keywords:** metal oxides, flame-spray pyrolysis, soft tissue, tissue repair, metals, nanoparticles, skin wounds, nanomedicine, wound healing, chronic wounds

## Abstract

Wound care and soft tissue repair have been a major human concern for millennia. Despite considerable advancements in standards of living and medical abilities, difficult-to-heal wounds remain a major burden for patients, clinicians and the healthcare system alike. Due to an aging population, the rise in chronic diseases such as vascular disease and diabetes, and the increased incidence of antibiotic resistance, the problem is set to worsen. The global wound care market is constantly evolving and expanding, and has yielded a plethora of potential solutions to treat poorly healing wounds. In ancient times, before such a market existed, metals and their ions were frequently used in wound care. In combination with plant extracts, they were used to accelerate the healing of burns, cuts and combat wounds. With the rise of organic chemistry and small molecule drugs and ointments, researchers lost their interest in inorganic materials. Only recently, the advent of nano-engineering has given us a toolbox to develop inorganic materials on a length-scale that is relevant to wound healing processes. The robustness of synthesis, as well as the stability and versatility of inorganic nanotherapeutics gives them potential advantages over small molecule drugs. Both bottom-up and top-down approaches have yielded functional inorganic nanomaterials, some of which unite the wound healing properties of two or more materials. Furthermore, these nanomaterials do not only serve as the active agent, but also as the delivery vehicle, and sometimes as a scaffold. This review article provides an overview of inorganic hybrid nanotherapeutics with promising properties for the wound care field. These therapeutics include combinations of different metals, metal oxides and metal ions. Their production, mechanism of action and applicability will be discussed in comparison to conventional wound healing products.

## 1. Introduction

The first injury treatments were described 5 millennia ago, when hunter-gatherers noticed that environmental factors and certain herbal remedies would speed up the wound healing process [1]. In ancient history, humans recognized the necessity of hygiene and the halting of bleeding, which led to the development of wound dressing techniques and suturing [2]. Despite the large number of general technological advancements over the past 100 years, progress beyond ancient wound care practices is surprisingly modest [3]. Apart from the introduction of antibiotics, and the ability to engineer tissues, modern wound management techniques only marginally differ in their core from the ones used in ancient times.

Wound healing complications remain responsible for an unacceptably high rate of morbidity and mortality. Reasons for that include the intrinsic vulnerability of the healing process, in conjunction with the lack of a holistic understanding of wound healing and the increasing occurrence of antimicrobial-resistant microbes [4]. Based on UK numbers, the estimates are that ~5% of the population in developed countries suffer from a wound, and ~40% of those patients have wound healing complications [5]. Adjusted for comorbidities, the average additional spending on wound management per patient is USD 3.5 K. In the USA, around 15% of Medicare beneficiaries suffer from a wound or wound infection [6,7], generating average costs of up to USD 11.8 K per patient. Evidence shows that these costs will rise in the upcoming years due to the aging population and the increasing occurrences of chronic conditions such as diabetes [5,8].

Soft tissue repair after trauma or surgery is an extremely delicate process that involves a whole cascade of events, namely, hemostasis, inflammation, angiogenesis and wound remodeling. Perturbation of any of these steps in the healing cascade can lead to severe consequences, ranging from unsatisfactory aesthetic outcome to patient death from bleeding or bacteremia. Wounds are generally classified according to their healing time; acute wounds heal themselves in a predictable time frame, whereas chronic wounds do not heal, leading to further complications, such as infection and necrosis. Acute wounds typically originate from surgical interventions or trauma. Chronic wounds, on the other hand, arise due to poor blood circulation, bacterial infection or systemic illnesses [9].

There is a variety of wound care products available on the global USD 25 billion market [10]. While some products focus on initial skin closure and sealing, others focus on longer-term wound therapy. In the first group, apart from suturing and stapling, there are several tissue adhesives for wound management. The commercially available products include cyanoacrylate-based glues (such as Dermabond), purified bovine serum albumin and glutaraldehyde (Bioglue), fibrin (such as Tisseel and Tachosil) and synthetic polymer sealants (e.g., TissuGlu). However, the available surgical adhesives often suffer from low adhesion, inappropriate mechanical strength, cytotoxicity concerns and poor performance in biological environments [11].

For prolonged wound therapy, various wound dressing materials are available on the market to support innate wound healing, such as alginate and collagen dressings. Most recently, bioengineered tissue constructs have found their way into clinics with promising results [12]. Such products include epidermal and dermal substitutes, such as Epicel and Integra. Despite significant technological advances, difficult-to-manage wound healing complications pose constitutive challenges to healthcare professionals. While wound care has undergone several transformative revolutions, hallmarked by the discovery of antibiotics which was followed by the ability to engineer tissues and tissue substitutes, conceptionally new approaches have entered clinics comparatively slowly [13]. A holistic wound care solution that covers the healing process from closure to scarring is yet to be found. Ideally, such a solution should be easily applicable and support the different phases of wound healing, while keeping microbes and excessive myofibroblast invasion (causative of scarring/fibrosis) at bay [13]. Recently, inorganic nanoparticles have been proven to be prime candidates for wound closure and management. As opposed to biologically derived agents, inorganic synthesis processes are usually easily scalable, inexpensive, robust, and allow a high degree of control over material architecture. The bioactive materials incorporated in such nanoparticles can be beneficial to a plethora of wound healing processes, and have anti-infectious properties. Remarkably, inorganic nanoparticles can be engineered to be intrinsically bioactive, thus uniting the drug and the carrier. This review describes important concepts in wound healing, and presents current and future solutions for wound management. The role of hybrid inorganic nanotherapeutics in the field is discussed, and their advantages, limitations and future prospects are examined.

## 2. Skin Wound Therapy and Clinical Needs

### 2.1. Phases of Wound Healing

**Figure 1:**
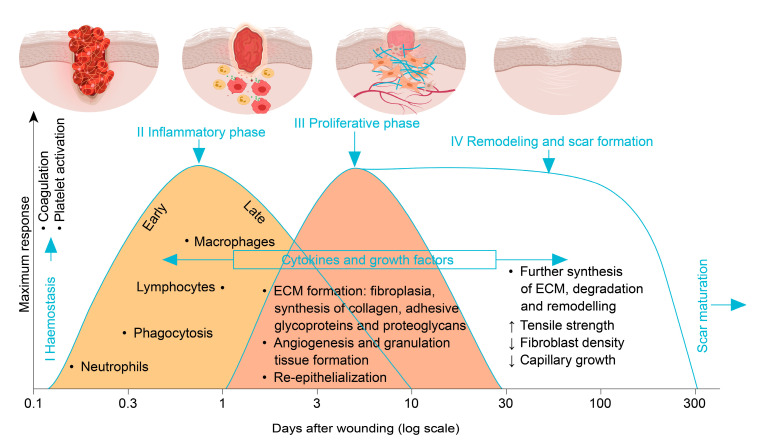
Injury and phases of wound healing. Wound healing timeline with important processes and actors (adapted from Enoch and Leaper [14] with permission from Elsevier).

Wound healing involves intricate interactions between various cell types, coagulation factors, connective tissue, growth factors, cytokines and the vascular system. Generally, the wound healing process can be divided into four stages, as illustrated in Figure 1 [14]. Within the first few minutes of injury, platelets in the blood begin to stick to the injured site. This results in the activation of fibrin, which then forms a mesh and acts as a glue to bind platelets to each other. The resulting clot then serves to plug the leak in the blood vessel, preventing further bleeding (Step 1: Hemostasis). Once hemostasis has been achieved, blood vessels dilate to allow essential cells, proteins and nutrients to reach the site of injury. The predominant cells at work here are the phagocytic cells mounting a host response and autolyzing any devitalized necrotic tissue (Step 2: Inflammation) [15]. During proliferation, the wound is then filled with new granulation tissue, which is comprised of extracellular matrix (collagen, etc.) and in which a new network of blood vessels develops (Step 3: Angiogenesis). The integrity of granulation tissue is critically dependent on fibroblasts receiving sufficient levels of oxygen and nutrients supplied by the newly-formed blood vessels. Maturation is the final phase, which occurs once the wound has closed and involves the remodeling of collagen (Step 4: Remodeling). The inappropriate execution of any of the above steps may critically affect the wound healing process and its success. In case the native wound healing capacity proves insufficient (see the sections below for possible reasons), therapeutic interventions become necessary. Additionally, the prevention of wound infection is of paramount importance, especially at times when antimicrobial resistances are on the rise.

### 2.2. Current Routine Measures for Skin Wound Management

**Figure 2:**
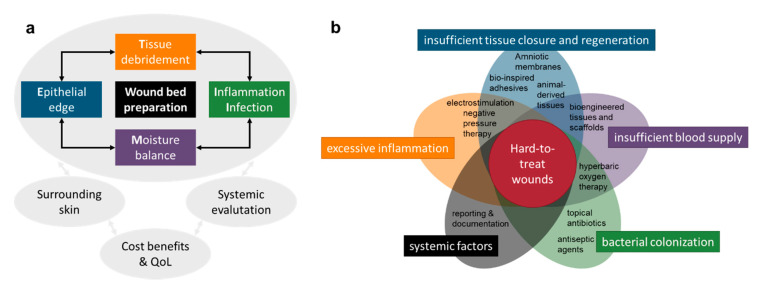
(**a**) Patient evaluation based on the extended TIME (tissue, infection/inflammation, moisture balance and edge of wound) principle. Adapted from [21] with permission from International Wound Journal, Blackwell Publishing Ltd. and Medicalhelplines.com Inc. (**b**) Main causes of the development of problematic wounds and currently used clinical measures to combat them. QoL: Quality of Life.

Current management strategies for chronic wounds focus on the treatment of the underlying causes, as well as the application of local treatments that allow wound healing mechanisms to take place in an optimal healing environment. As illustrated in Figure 2a, these local treatments usually include (i) measures to remove slough, debris and cells that impair healing (surgical, mechanical or chemical debridement), (ii) the control of the bacterial burden and inflammation with antiseptic substances if needed and (iii) the use of dressings that provide an ideal moisture balance to facilitate healing processes. However, for many of these measures, there is a lack of sound evidence for their effectivity [16,17]. Despite considerable efforts, many wounds take a long time to heal, which further aggravates the disease burden for the individual patient as well as the economic burden for the society.

### 2.3. Disease and Other Factors Affecting Skin Wound Healing

Generally, wound healing can be affected by systemic factors that bear little or no direct relation to the wound itself (age, body type, chronic diseases, etc.), and local factors concerning the wound itself (desiccation, abnormal bacterial presence, trauma, etc) [18]. Wounds that show a prolonged healing time are usually stuck in the inflammatory phase of wound healing. This is a hallmark of chronic wounds, and is usually caused by an underlying disease impairing blood supply, such as macrovascular disease (chronic venous insufficiency, peripheral arterial occlusive disease), or chronic pressure reducing microvascular blood flow (diabetic foot ulcers, pressure ulcers) [19]. Wound infection can also prolong the inflammatory phase of wound healing, and it can result both from hospital-acquired or other external germs, as well as from the patient’s flora. Furthermore, there is a large number of systemic diseases and risk factors that can impair wound healing processes by additionally reducing blood supply and oxygenation (e.g., smoking, diabetes mellitus), and impairing macrophage function, growth factor production and other reparative processes (e.g., diabetes mellitus, malnutrition) [20].

### 2.4. Clinically Used Measures to Support Healing of Problematic Wounds

Figure 2b summarizes the factors leading to problematic wounds, and current measures taken to treat them. Supportive measures, such as physical therapies (including electrostimulation and shock-waves), negative pressure and hyperbaric oxygen therapies, can stimulate wound healing, and have attracted significant attention [1]. Besides, growth factors therapies have been intensively researched in the last few decades. However, the success of growth factor therapies in double-blinded randomized controlled trials has been limited [22]. The delivery of the exogenous growth factors remains a major challenge, and recent research has therefore increasingly shifted to alternative delivery approaches [23]. More sophisticated bioengineered materials have been developed recently, which hold promise with regard to accelerating wound healing processes. Products based on porcine intestine or fish skin have been shown to facilitate wound healing by providing a scaffold for the regenerating cells, as well as modifying the wound healing environment. Amniotic membranes can be used as a potent source of growth factors [24]. Tissue-engineered dermal and epidermal substitutes, or combinations thereof, have also been shown to provide cells needed in the wound healing process, and modify cytokines and growth factors in wounds, and thus speed up wound healing [25]. However, the relatively high costs of these products and a variety of regulatory issues have so far prevented their widespread clinical use [26].

### 2.5. Clinical Needs and Challenges

Over the last few decades, the understanding of wound healing has increased immensely due to technological advancement, and a growing body of clinical and scientific research. Despite all this progress, non-healing wounds still pose a massive burden on patients and the healthcare system. While some patients receive inadequate treatment due to insufficient clinical reporting and the lack of reimbursement for effective treatments [6], current gold-standard measures still fail to heal many types of problematic wounds. Infections, insufficient closure, perfusion and the healing of the wounds still pose intricate problems to health care professionals. An ideal future wound care product should be applicable to any wound type, any depth and any exudate amount, and will eradicate biofilms, control wound odor, reduce pain and reduce treatment time, all while being cost-effective. Additionally, it should be usable in different care settings. For this goal, such a product should combine the diverse features of contemporary developments in a single composite wound dressing.

## 3. Emerging Skin Wound Care Materials

Despite the sometimes slow transition to clinics, research into wound care is very active, and novel approaches and concepts are published frequently. In this section, we will discuss recent conceptually novel approaches to wound management, as well as their opportunities and drawbacks.

### 3.1. Bioengineered Tissues and Scaffolds

The rapid development of molecular biology and biotech has enabled engineers to design functional tissues and scaffolds using a combination of synthetic and biological materials. Tissue bioengineering aims to substitute, restore, or improve compromised human tissue. Commonly, the body’s own healing capacities are harnessed and guided by the artificial tissue [27,28].

**Figure 3:**
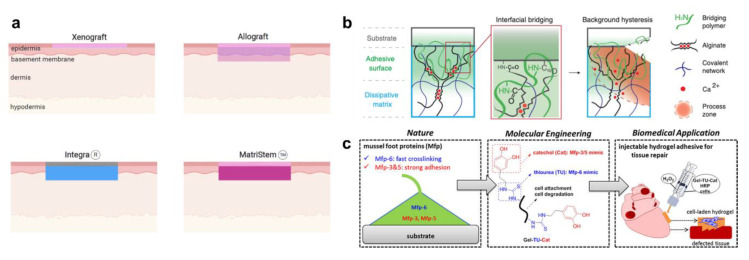
(**a**) Different wound covers and regeneration templates [30]. (**b**) Dual-layer hydrogel adhesives inspired by slugs. From [36]. Reprinted with permission from the American Association for the Advancement of Science (**c**) Injectable adhesives inspired by mussel foot proteins. Reprinted with permission from [37]. Copyright 2019 American Chemical Society.

In particular, the treatment of skin wounds with engineered tissues has seen rapid development in the past years, with several solutions gaining market access (Figure 3a). On the one hand, acellular products such as Integra and Alloderm are composed of synthetic materials and cell-free biologically derived components such as collagen. On the other hand, allogeneic skin substitutes, such as Apligraf and Dermagraft, consist of cell-layers seeded onto a biomaterial scaffold. Most recently, patient biopsies have been used to culture skin autografts from their own cellular material. Currently, Epicel is the only approved product of that kind [29], but other solutions such as Permaderm and denovoSkin are on their way to clinics [30]. Such products have the benefit of using a patient’s own material for healing, but require invasive biopsies and extended culturing times [27].

Despite promising results and market-approved products, tissue engineering plays a comparably small role in patient treatment [31]. This limited use is mostly due to the experimental nature of the treatments and the high costs associated with them [28]. A big concern in the field is the limited reproducibility of complex artificial tissue, due to the insufficient control of the underlying biological processes. Additionally, ethical concerns have been raised about the use of animal- or human-derived materials, and the invasiveness of tissue sampling from the patient [32,33].

### 3.2. Bioinspired Materials for Wound Care

Over billions of years of evolution, nature has come up with brilliant solutions for the obstacles that a variety of organisms have faced. In the past decades, material researchers have tapped into this wealth of innovation and tried to mimic some of nature’s approaches [34,35]. Various animals have been mimicked to overcome wound care issues. Li et al. [36] reported on a slug-inspired design for adhesives consisting of two polymer layers: an adhesive layer and a dissipative matrix. The adhesive layer adheres to the substrate by electrostatic interactions, covalent bonds and infiltration into the tissue. The dissipative layer amplifies energy dissipation through hysteresis. In this way, they achieved strong adhesion even on wet surfaces (Figure 3b). Mussels are a common source of inspiration due to their unique stickiness [37,38,39] (Figure 3c) and the structural benefits of nacre [40]. Further, the properties of geckos [41] and spider webs [42] have been imitated to achieve strong adhesive properties in materials. Lee et al. [43] drew inspiration from sandcastle worms, and devised a light-activated adhesive based on poly(glycerol sebacate)-acrylate nanoparticles. The adhesive is injectable into tissue due to its low initial viscosity, and has been shown to withstand considerable forces after curing. Last but not least, humans have also been used for inspiration by Blacklow et al. [44], who have tried to mimic embryonic wound closure by mechanical stimuli, and by Annabi et al. [45], who have harnessed the elastic properties of human tropoelastin to engineer an elastic sealant. While the aforementioned approaches to novel materials are promising for tissue closure, more development is needed to address the later stages of wound healing and treatment.

### 3.3. Metal and Metal Oxide Nanoparticles for Skin Wound Care

With the push to simplify materials designed for healthcare applications, inorganic nanomaterials have re-gained interest. The controlled synthesis of nanoparticles (NPs) is arguably one of the most important achievements of material science in the past decades [46]. The emergence of nanotechnology has extended the toolset of biomaterial research, with stable materials that are transportable by the body and have a large interaction surface. Inorganic, and especially metal and metal oxide NPs, have attracted significant attention due to their scalability and robustness of synthesis, low costs, and tailorable architecture [46,47]. Consequently, they have been used as MRI contrast agents [48], radio enhancers [49], for blood purification [50,51] and for photodynamic therapy [52].

**Figure 4:**
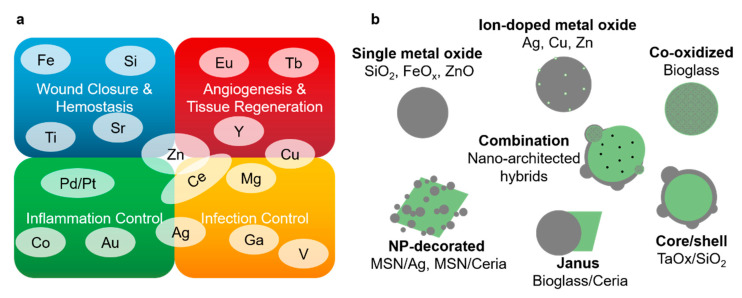
(**a**) Overview of the use of metal oxides in nanoparticulate form for wound healing related processes [59,60,72,73,74,75,76]. (**b**) Different possible architectures and structures of metal oxide nanohybrids.

Recent advances in nanotechnology, in combination with an increased understanding of the pathophysiology of wounds, have resulted in the development of several inorganic materials and fillers to assist wound healing (Figure 4a) [53,54,55]. While fascinating results have been achieved by loading inorganic nanoparticles with organic molecules [56,57], the following sections are focused on purely inorganic nanosystems. To mitigate inflammation, for instance, the antioxidant properties of gold [58], titanium [59], palladium and platinum [60] oxide NPs have been harnessed. For infection control [61,62], wound dressings with silver NPs have been used for decades [63,64], however toxicity and resistance concerns [65] have shifted the focus to alternative materials such as ZnO [66] or CeO_2_ [67]. Cerium oxide-based nanoparticles have further been shown to decrease oxidative stress and support cell proliferation [68]. 

As a novel approach to tissue closure, Leibler and colleagues [69,70] have introduced the concept of nano-bridging—tissue adhesion achieved by a mere physical phenomenon. They topically applied aqueous suspensions of silica and iron oxide to connect biological tissues by forming an adhesive layer between them. The nanoparticles act as connectors between macromolecules, which then serve as bridges between different particles, eventually leading to the adhesion and macroscopic gluing of tissue. As a result, a bridging layer of NPs is formed that can sustain high amounts of stress due to rearrangement processes on the particle surface. The topical application of silica and iron oxide nanoparticles, as suggested by Leibler and colleagues, may indeed be an elegant way to rapidly achieve hemostasis and wound closure. Kim et al. [71] achieved an increased adhesive effect by augmenting the silica NP surface area. While the advantages of silica and iron oxide NPs compared to current wound closing techniques have been demonstrated, the particles were solely tested for their adhesive properties, neglecting their possible bioactivity and compatibility. There is a plethora of metal oxides that can advance or mitigate specific wound healing processes in their nanoparticulate form. To achieve successful wound healing, however, multiple stages of the wound healing cascade have to be addressed. A simple yet elegant approach to addressing different processes with multiple materials is to unite beneficial properties in the form of hybrid NPs (Figure 4b).

## 4. Metal Oxide Nanoparticle Hybrid Materials

As described in the previous section, a variety of metal oxide NPs show great promise for wound healing applications [54]. To unite different bioactive properties in one therapeutic agent, or to amplify one of them, the logical progression is to combine materials in hybrid nanoparticles. Such a multi-component approach benefits from the fact that the individual component activity can be tailored independently to the application, provided that there is no interference between the different material components. The vast number of possible material combinations and architectures gives access to a large design space, and hence properties can be accurately tailored to the intended application and specific clinical needs in a precision medicine-like approach. The increase in the complexity of such nanotherapeutics, however, counterbalances the straightforwardness and robustness of inorganic materials. This is why reproducible and stable synthesis methods are key. Ideally, the metal oxide nanohybrids are produced in a few steps, and do not require post-synthesis modification. While large parts of the nanohybrid research have focused on the organic post-modification of inorganic nanoparticle carriers to achieve multi-functionality, this section is focused exclusively on inorganic hybrid nanotherapeutics. In contrast to complex carrier-based drug release systems, therapeutics based on metal and metal oxides feature inherent bioactivity, effectively combining carrier and drug in one entity. In the past decade, a variety of inorganic hybrid nanotherapeutics have been developed and evaluated for wound care applications. Their different architectures and structures are summarized in Figure 4b, and selected examples are discussed in more detail below.

### 4.1. Blends of Metal Oxides

Several bioactive glasses based on borate, phosphate and silicate compositions have raised significant clinical interest in the past 50 years [77]. The first and most notable example of such a composition is Bioglass 45S5 (45 wt. % SiO_2_, 24.5 wt. % CaO, 24.5 wt. % Na_2_O and 6.0 wt. % P_2_O_5_), developed by Larry Hench in 1969 [78]. Initially mostly used for bone repair due to its unique biomineralization upon implantation, nanoparticulate bioglass and its variations have recently shown great promise in soft tissue wound healing [79]. Apart from its tissue bonding and hemostatic properties [80,81], some modifications, such as strontium-substitution [82], have been shown to accelerate cell proliferation and angiogenesis. Furthermore, several borate-based formulations have enhanced wound closure and human cell proliferation. Human wound healing is complex and so is the impact of bioactive inorganics on it. Due to the variety of metal oxides used, mechanisms and pathways are difficult to elucidate. It has been hypothesized that the enhanced soft tissue healing effect stems from the release of ions, as opposed to biomineralization, in bone defect healing [83]. Despite its mechanistic complexity, the wound healing activity of bioglass-based products in a wide variety of in vitro and in vivo models has proven to be highly robust.

### 4.2. Metal Oxide Nanoparticles Doped with Metal Ions

**Figure 5:**
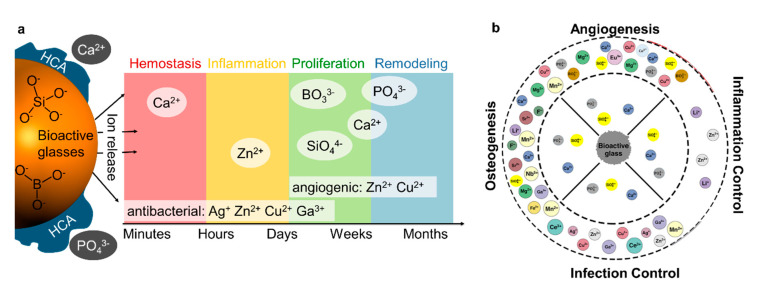
(**a**) Different dissolution products of borate- and silicate-based bioactive glasses and their effects on the wound healing cascade. (**b**) Bioglass dopants and their benefits in tissue healing. Inner ring: native silicate bioglass dissolution products. Outer ring: investigated dopants and their benefits for wound care. Copyright © 2020 Kargozar, Mozafari, Hamzehlou and Baino [84].

In particular, the aforementioned bioactive glasses have been used as a matrix for the controlled release of metal ions [84,85]. Figure 5a illustrates the effects of different dissolution products of doped bioactive glasses on the wound healing cascade. A wide spectrum of ion dopants has been investigated for beneficial properties related to wound healing and tissue repair (Figure 5b).

Furthermore, a variety of single-metal oxide nanoparticulate matrices were doped to enhance their use for wound care. For instance, antimicrobial Ag NPs doped with Zn accelerated wound healing in rats [86], while the Zn-doping of CuO NPs increased their antimicrobial activity [87]. Several research groups enhanced the antibacterial properties of multifunctional ZnO NPs by doping with Co [88], Mn [89] and Ta [90]. In another instance, TiO_2_ NPs doped with Zn showed pro-angiogenic properties [91], while doping with Cu yielded antibacterial properties [92].

### 4.3. Inorganic Frameworks Decorated With Metal Oxide Nanoparticles

Instead of ion doping a framework, it can be decorated with nanoparticles composed of the same material, giving further control over release kinetics. The commonly used nano-sized frameworks for NP-decoration are mesoporous silicate networks (MSNs).

Such a framework has been decorated with ferrite and ceria nanoparticles to yield strong anti-inflammatory properties [93]. Similarly, adhesive MSNs have been decorated with cerium oxide to quench ROS, and thus locally downregulate inflammation [94]. Jin et al. [95] have designed Ag/AgBr-loaded MSNs with antimicrobial properties and potential wound healing properties. Furthermore, Ag nanoparticles have been decorated with Fe_3_O_4_ and MnO_2_ to improve the stability, biocompatibility and availability of Ag ions. To enhance the already discussed anti-inflammatory activity of ceria nanoparticles, Munusamy et al. stabilized their oxidation state by immobilization on silica supports [96].

### 4.4. Core/shell and Janus-Shaped Hybrids

As structure dictates function, tuning the architecture of a nanohybrid can have a large impact on its biomedical properties. A system that has shown promising adhesive properties is the tantalum oxide/silica core/shell nanoparticles [97]. As an additional benefit, these are detectable by a range of imaging methods, such as ultrasound or x-ray, and can be harnessed as fiducial markers for image-guided interventions. More sophisticated architectures have been proposed, including multiple shell structures such as the Fe_3_O_4_@SiO_2_@Ag@porousSiO_2_ disinfectant nanoparticles proposed by Wang et al. [98]. Another instance of a sophisticated antimicrobial system has been investigated by Zhu et al. [99], which consists of silver nanoparticles-decorated and mesoporous silica-coated single-walled carbon nanotubes. Recently, He et al. [100] performed the synthesis of bioactive zeolitic imidazolate framework-8-capped ceria nanoparticles (CeO_2_@ZIF-8 NPs) in order to achieve enhanced catalytic and antioxidative activities. They claim that their hybrid system overcomes the drawbacks of pure CeO_2_ by the following mechanisms: (i) ZIF-8 acting as peroxidase to maintain the antioxidant activity in the presence of excessive H_2_O_2_ or other oxidants; (ii) enabling control of the size, shape and surface charge of the CeO_2_ core by ZIF-8; (iii) releasing active components during ZIF-8 degradation, which synergistically enhances the efficacy of CeO_2_.

### 4.5. Combinations of the Above: Nano-Architected Hybrids

**Figure 6:**
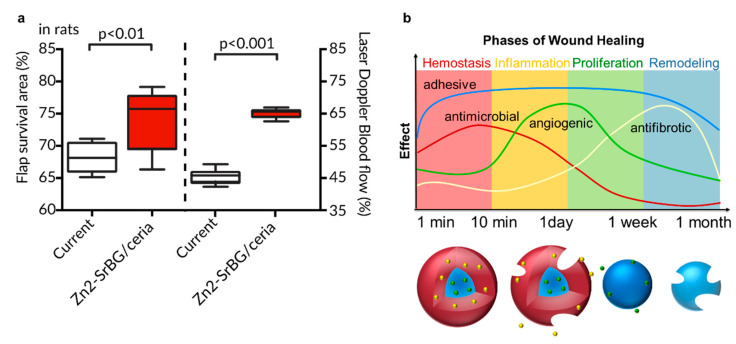
(**a**) A combined architectural approach, i.e., Janus-shaped hybrid nanoparticles made out of cerium oxide and Zn-doped Sr-substituted bioglass increased skin flap survival in a rat model. The increased survival area was most likely due to the angiogenic properties of the nanoparticles and the resulting higher tissue perfusion (adapted from Lese at al. [108]). (**b**) By engineering the architecture and structure of a metal oxide nanotherapeutic, the temporal control of material activity can be achieved. This control allows the synchronization of the desired effect to the desired time.

Recent studies by us have reported the first results regarding the potential of uniting adhesion, based on nano-bridging, with bioactivity [101]. Uniting these properties requires control over nanoparticle architecture and freedom of choice in materials. Liquid-feed flame spray pyrolysis (LF-FSP) fulfills these requirements, while additionally enabling one-step and high-throughput synthesis [102]. By utilizing the versatility of LF-FSP, we have united the wound closure properties of bioglass with the anti-inflammatory properties of ceria in one nanoparticle hybrid. That hybrid showed strong adhesiveness, fast hemostasis and low cytotoxicity [101]. Furthermore, we were able to control the oxidation state of the ceria portion of said hybrid, which governs its anti-oxidative and anti-microbial properties [103]. In the next step, we adapted the hybrids to be more beneficial in a soft tissue healing scenario by substituting part of the Ca portion in the bioglass matrix with Sr, and doping it with Zn. Both elements and their oxides have shown anti-inflammatory and, more importantly, angiogenic properties [104,105,106,107]. In a perforator flap study in rats, these nanoparticles significantly increased healing, most probably due to an increased perforation of the skin flap (Figure 6a) [108]. To address the phases of the wound healing cascade in an ideal way, temporal control of the material activities is pivotal. By tailoring the architecture of the hybrid nanoparticles, we believe that such control can be reached, and therapeutic performance can be optimized (Figure 6b).

## 5. Disadvantages and Dangers

Despite the promising effects of inorganic hybrid nanotherapeutics, there are toxicity concerns, as with every new technology. The scientific community and regulatory agencies, including the Food and Drug Administration (FDA), have recently intensified discussions regarding the safety concerns of inorganic nanomaterials and their application to intact skin (cosmetic products) [109,110]. Nanomaterial application to areas where the natural barrier integrity has been compromised (e.g., wounds) will lead to a more direct exposure to the nanomaterials. Nanoparticle biodistribution and biological effects, including toxicity, are determined by a multitude of parameters of varying importance, such as material composition, particle size, shape and charge [111]. The properties of nanomaterials and their behavior are distinctly different from small molecules and bulk materials [112], hence requiring the tailoring of established safety and toxicity assessment procedures. The Nanotechnology Characterization Labs (NCL) have been established in the US (NCL [113]) and EU (EU-NCL [114]), offering guidance regarding the safety and toxicity assessment of nanomaterials for clinical applications. With the increasing availability of nanomaterial toxicity data, safe-by-design approaches are gaining increasing attention [115,116]. Commercially available inorganic nanoparticle-based wound care products, such as silver-based formulations, are widely used in the management of chronic wounds and infections. However, the application of these inorganic nanoparticle-based wound care products is limited, owing to toxicity concerns as well as long-term tissue deposition (argyrosis). Additionally, granuloma formation has occasionally been observed following the application of inorganic nanoparticles to wounds [117]. Issues such as long-term tissue deposition and the degradation of the inorganic nanoparticles compromise their application for human use. Without a proper understanding of the long-term fate of nanoparticles in biological systems [118], their clinical translation becomes critical [119,120,121,122,123].

The use of high-dose non-degradable metal oxide nanoparticles may significantly limit the clinical applicability of the procedure, due to the high stability of the nanoparticles and the associated unknown risks (including off-target effects, inflammation, reactive oxygen species formation and re-distribution to reticuloendothelial system and organs). For a safe translation, an in-depth understanding of nanoparticle biodistribution and biotransformation is imperative. Recent studies by us have reported the multiscale, multimodal characterization of inorganic nanohybrids in rats using a cascade of advanced microscopy and spectroscopy techniques. The study revealed that when inorganic nanohybrids are intravascularly injected, they expectedly accumulate in the liver, spleen and kidney. When applied topically to the subcutis, nanohybrids accumulate in tissue-resident macrophages, and no significant re-distribution to organs other than the skin could be observed for observation periods of one week [124]. These findings, and the absence of any detectable systemic effects, indicate that topical application may indeed give access to some of the unique potential metal oxide nanotechnology has to offer to the wound healing field. However, inorganic nanoparticles are not straightforward to apply to tissue. Dry powders have unpredictable aggregations, are difficult to apply evenly, and can become airborne. Upon dispersion in aqueous media, however, the inorganic nanoparticle suspensions often sediment over time, again rendering controlled application more challenging. Nonetheless, promising clinically relevant actions of inorganic nanotherapeutics have already been demonstrated, despite the current challenges associated with their delivery.

## 6. Conclusions and Future Prospects

Skin wounds are a growing burden for clinicians and the economy due to an aging society and the rise of vascular diseases, diabetes and antibiotic resistance. The current wound treatment success with conventional wound care measures is limited due to several factors, such as bacterial infection, insufficient blood supply and excessive inflammation. Novel approaches, such as biologically derived and bio-inspired materials, have shown promising results, but are limited to specific use cases and are usually accompanied by high costs. Bioinspired polymers demonstrate great potential as adhesives, but have limited use in long-term wound healing. While there are many novel approaches to wound healing, inorganic materials represent one of the most promising ones, due to their low-immunogenicity, versatility, robustness and scalability. By utilizing inherently bioactive metal oxides, drug and carrier can be united in one nanotherapeutic agent. The combination of such bioactivities, such as anti-infectious, anti-inflammatory and adhesive properties, can be achieved in metal oxide hybrid nanoparticles. Such a combination allows the tailoring of the desired effects to different stages of the wound healing cascade. Ideally, temporal control of the material properties is achieved, making sure that the right effect takes place at the right time. Several steps in that direction have been made, and the scientific community is not far from reaching that goal. 

## References

[1] Han G., Ceilley R. (2017). Chronic Wound Healing: A Review of Current Management and Treatments. Adv. Ther..

[2] Sarabahi S. (2012). Recent advances in topical wound care. Indian J. Plast. Surg..

[3] Shah J.B. (2012). The History of Wound Care. J. Am. Coll. Certif. Wound Spec..

[4] Basu S., Shukla V., Mani R., Romanelli M., Shukla V. (2012). Complications of Wound Healing. Measurements in Wound Healing.

[5] Guest J.F., Ayoub N., McIlwraith T., Uchegbu I., Gerrish A., Weidlich D., Vowden K., Vowden P. (2015). Health economic burden that wounds impose on the National Health Service in the UK. BMJ Open.

[6] Nussbaum S.R., Carter M.J., Fife C.E., DaVanzo J., Haught R., Nusgart M., Cartwright D. (2018). An Economic Evaluation of the Impact, Cost, and Medicare Policy Implications of Chronic Nonhealing Wounds. Value Health.

[7] 2015 Edition | CMS. https://www.cms.gov/Research-Statistics-Data-and-Systems/Statistics-Trends-and-Reports/CMS-Statistics-Reference-Booklet/2015.

[8] Gillespie P., Carter L., McIntosh C., Gethin G. (2019). Estimating the health-care costs of wound care in Ireland. J. Wound Care.

[9] Frykberg R.G., Banks J. (2015). Challenges in the Treatment of Chronic Wounds. Adv. Wound Care.

[10] Wound Care Market—Forecast to 2024 | Growing at a CAGR of 4.6% | MarketsandMarkets. https://www.marketsandmarkets.com/Market-Reports/wound-care-market-371.html.

[11] Jain R., Wairkar S. (2019). Recent developments and clinical applications of surgical glues: An overview. Int. J. Biol. Macromol..

[12] Savoji H., Godau B., Hassani M.S., Akbari M. (2018). Skin Tissue Substitutes and Biomaterial Risk Assessment and Testing. Front. Bioeng. Biotechnol..

[13] Dhivya S., Padma V.V., Santhini E. (2015). Wound dressings—A review. Biomedicine.

[14] Enoch S., Leaper D.J. (2005). Basic science of wound healing. Surgery.

[15] Rodrigues M., Kosaric N., Bonham C.A., Gurtner G.C. (2018). Wound Healing: A Cellular Perspective. Physiol. Rev..

[16] Fife C.E., Carter M.J., Walker D. (2010). Why is it so hard to do the right thing in wound care?. Wound Repair Regen..

[17] Franks P.J., Barker J., Collier M., Gethin G., Haesler E., Jawien A., Laeuchli S., Mosti G., Probst S., Weller C. (2016). Management of Patients With Venous Leg Ulcers: Challenges and Current Best Practice. J. Wound Care.

[18] Armstrong D.G., Meyr A.J. (2020). Risk factors for impaired wound healing and wound complications. UpToDate.

[19] Falanga V. (2005). Wound healing and its impairment in the diabetic foot. Lancet.

[20] Anderson K., Hamm R.L. (2012). Factors That Impair Wound Healing. J. Am. Coll. Clin. Wound Spec..

[21] Leaper D.J., Schultz G., Carville K., Fletcher J., Swanson T., Drake R. (2012). Extending the TIME concept: what have we learned in the past 10 years?. Int. Wound J..

[22] Martí-Carvajal A.J., Gluud C., Nicola S., Simancas-Racines D., Reveiz L., Oliva P., Cedeño-Taborda J. (2015). Growth factors for treating diabetic foot ulcers. Cochrane Database Syst. Rev..

[23] Catanzano O., Boateng J., Boateng J. (2020). Local Delivery of Growth Factors Using Wound Dressings. Therapeutic Dressings and Wound Healing Applications.

[24] Litwiniuk M., Grzela T. (2014). Amniotic membrane: New concepts for an old dressing. Wound Repair Regen..

[25] Dussoyer M., Michopoulou A., Rousselle P. (2020). Decellularized Scaffolds for Skin Repair and Regeneration. Appl. Sci..

[26] Piaggesi A., Låuchli S., Bassetto F., Biedermann T., Marques A., Najafi B., Palla I., Scarpa C., Seimetz D., Triulzi I. (2018). Advanced therapies in wound management: cell and tissue based therapies, physical and bio-physical therapies smart and IT based technologies. J. Wound Care.

[27] Hoffman T., Khademhosseini A., Langer R. (2019). Chasing the Paradigm: Clinical Translation of 25 Years of Tissue Engineering. Tissue Eng. Part A.

[28] O’Donnell B.T., Ives C.J., Mohiuddin O.A., Bunnell B.A. (2019). Beyond the Present Constraints That Prevent a Wide Spread of Tissue Engineering and Regenerative Medicine Approaches. Front. Bioeng. Biotechnol..

[29] Sundaram S., Siewert J., Balestrini J., Gard A., Boehm K., Wilcox E., Niklason L., Simon T.L., McCullough J., Snyder E.L., Solheim B.G., Strauss R.G. (2016). Tissue engineering and regenerative medicine. Rossi’s Principles of Transfusion Medicine.

[30] Adibfar A., Retrouvey H., Padeanu S., Jeschke M.G., Shahrokhi S. (2019). Current State of Selected Wound Regeneration Templates and Temporary Covers. Curr. Trauma Rep..

[31] Kim Y.S., Smoak M.M., Melchiorri A.J., Mikos A.G. (2019). An Overview of the Tissue Engineering Market in the United States from 2011 to 2018. Tissue Eng. Part A.

[32] King N.M.P., Coughlin C.N., Furth M. Ethical Issues in Regenerative Medicine. https://ssrn.com/abstract=1380162.

[33] Chan S. (2017). Current and emerging global themes in the bioethics of regenerative medicine: the tangled web of stem cell translation. Regen. Med..

[34] Suarato G., Bertorelli R., Athanassiou A. (2018). Borrowing From Nature: Biopolymers and Biocomposites as Smart Wound Care Materials. Front. Bioeng. Biotechnol..

[35] Green D.W., Ben-Nissan B., Yoon K.-S., Milthorpe B., Jung H.-S. (2016). Bioinspired materials for regenerative medicine: going beyond the human archetypes. J. Mater. Chem. B.

[36] Li J., Celiz A.D., Yang J., Yang Q., Wamala I., Whyte W., Seo B.R., Vasilyev N.V., Vlassak J.J., Suo Z. (2017). Tough adhesives for diverse wet surfaces. Science.

[37] Wei K., Senturk B., Matter M.T., Wu X., Herrmann I.K., Rottmar M., Toncelli C. (2019). Mussel-Inspired Injectable Hydrogel Adhesive Formed under Mild Conditions Features Near-Native Tissue Properties. ACS Appl. Mater. Interfaces.

[38] Chen W., Wang R., Xu T., Ma X., Yao Z., Chi B., Xu H. (2017). A mussel-inspired poly(γ-glutamic acid) tissue adhesive with high wet strength for wound closure. J. Mater. Chem. B.

[39] Sousa M.P., Neto A.I., Correia T.R., Miguel S.P., Matsusaki M., Correia I.J., Mano J.F. (2018). Bioinspired multilayer membranes as potential adhesive patches for skin wound healing. Biomater. Sci..

[40] Xue J., Wang X., Wang E., Li T., Chang J., Wu C. (2019). Bioinspired multifunctional biomaterials with hierarchical microstructure for wound dressing. Acta Biomater..

[41] Lee H., Lee B.P., Messersmith P.B. (2007). A reversible wet/dry adhesive inspired by mussels and geckos. Nature.

[42] Yuk H., Varela C.E., Nabzdyk C.S., Mao X., Padera R.F., Roche E.T., Zhao X. (2019). Dry double-sided tape for adhesion of wet tissues and devices. Nature.

[43] Lee Y., Xu C., Sebastin M., Lee A., Holwell N., Xu C., Miranda Nieves D., Mu L., Langer R.S., Lin C. (2015). Bioinspired Nanoparticulate Medical Glues for Minimally Invasive Tissue Repair. Adv. Healthcare Mater..

[44] Blacklow S.O., Li J., Freedman B.R., Zeidi M., Chen C., Mooney D.J. (2019). Bioinspired mechanically active adhesive dressings to accelerate wound closure. Sci. Adv..

[45] Annabi N., Zhang Y.-N., Assmann A., Sani E.S., Cheng G., Lassaletta A.D., Vegh A., Dehghani B., Ruiz-Esparza G.U., Wang X. (2017). Engineering a highly elastic human protein–based sealant for surgical applications. Sci. Transl. Med..

[46] Giner-Casares J.J., Henriksen-Lacey M., Coronado-Puchau M., Liz-Marzán L.M. (2016). Inorganic nanoparticles for biomedicine: where materials scientists meet medical research. Mater. Today.

[47] Andreescu S., Ornatska M., Erlichman J.S., Estevez A., Leiter J.C., Matijević E. (2012). Biomedical Applications of Metal Oxide Nanoparticles. Fine Particles in Medicine and Pharmacy.

[48] Issa B., Obaidat I.M. (2019). Magnetic Nanoparticles as MRI Contrast Agents. Magn. Reson. Imaging.

[49] Gerken L.R.H., Keevend K., Zhang Y., Starsich F.H.L., Eberhardt C., Panzarasa G., Matter M.T., Wichser A., Boss A., Neels A. (2018). Lanthanide-Doped Hafnia Nanoparticles for Multimodal Theranostics: Tailoring the Physicochemical Properties and Interactions with Biological Entities. ACS Appl. Mater. Interfaces.

[50] Herrmann I.K., Urner M., Koehler F.M., Hasler M., Roth-Z’Graggen B., Grass R.N., Ziegler U., Beck-Schimmer B., Stark W.J. (2010). Blood Purification Using Functionalized Core/Shell Nanomagnets. Small.

[51] Anthis A.H.C., Matter M.T., Keevend K., Gerken L.R.H., Scheibler S., Doswald S., Gogos A., Herrmann I.K. (2019). Tailoring the Colloidal Stability, Magnetic Separability, and Cytocompatibility of High-Capacity Magnetic Anion Exchangers. ACS Appl. Mater. Interfaces.

[52] Bechet D., Couleaud P., Frochot C., Viriot M.-L., Guillemin F., Barberi-Heyob M. (2008). Nanoparticles as vehicles for delivery of photodynamic therapy agents. Trends Biotechnol..

[53] Nethi S.K., Das S., Patra C.R., Mukherjee S. (2019). Recent advances in inorganic nanomaterials for wound-healing applications. Biomater. Sci..

[54] Urie R., Ghosh D., Ridha I., Rege K. (2018). Inorganic Nanomaterials for Soft Tissue Repair and Regeneration. Annu. Rev. Biomed. Eng..

[55] Silva M.M.P., de Aguiar M.I.F., Rodrigues A.B., Miranda M.D.C., Araújo M.Â.M., Rolim I.L.T.P., Souza A.M.A., Silva M.M.P., de Aguiar M.I.F., Rodrigues A.B. (2017). The use of nanoparticles in wound treatment: a systematic review. Revista da Escola de Enfermagem da USP.

[56] Miller K.P., Wang L., Benicewicz B.C., Decho A.W. (2015). Inorganic nanoparticles engineered to attack bacteria. Chem. Soc. Rev..

[57] Peña-Parás L., Sánchez-Fernández J.A., Vidaltamayo R., Martínez L.M.T., Kharissova O.V., Kharisov B.I. (2018). Nanoclays for Biomedical Applications. Handbook of Ecomaterials.

[58] Oueslati M.H., Tahar L.B., Harrath A.H. (2020). Catalytic, antioxidant and anticancer activities of gold nanoparticles synthesized by kaempferol glucoside from Lotus leguminosae. Arab. J. Chem..

[59] Agarwal H., Nakara A., Shanmugam V.K. (2019). Anti-inflammatory mechanism of various metal and metal oxide nanoparticles synthesized using plant extracts: A review. Biomed. Pharmacother..

[60] Shibuya S., Ozawa Y., Watanabe K., Izuo N., Toda T., Yokote K., Shimizu T. (2014). Palladium and Platinum Nanoparticles Attenuate Aging-Like Skin Atrophy via Antioxidant Activity in Mice. PLoS ONE.

[61] Mihai M.M., Dima M.B., Dima B., Holban A.M. (2019). Nanomaterials for Wound Healing and Infection Control. Materials.

[62] Mihai M.M., Preda M., Lungu I., Gestal M.C., Popa M.I., Holban A.M. (2018). Nanocoatings for Chronic Wound Repair—Modulation of Microbial Colonization and Biofilm Formation. Int. J. Mol. Sci..

[63] Paladini F., Pollini M. (2019). Antimicrobial Silver Nanoparticles for Wound Healing Application: Progress and Future Trends. Materials.

[64] Boateng J., Catanzano O. (2020). Silver and Silver Nanoparticle-Based Antimicrobial Dressings. Therapeutic Dressings and Wound Healing Applications.

[65] Chernousova S., Epple M. (2013). Silver as Antibacterial Agent: Ion, Nanoparticle, and Metal. Angew. Chem. Int. Ed..

[66] Chhabra H., Deshpande R., Kanitkar M., Jaiswal A., Kale V.P., Bellare J.R. (2016). A nano zinc oxide doped electrospun scaffold improves wound healing in a rodent model. RSC Adv..

[67] Reed K., Cormack A., Kulkarni A., Mayton M., Sayle D., Klaessig F., Stadler B. (2014). Exploring the properties and applications of nanoceria: is there still plenty of room at the bottom?. Environ. Sci. Nano.

[68] Chigurupati S., Mughal M.R., Okun E., Das S., Kumar A., McCaffery M., Seal S., Mattson M.P. (2013). Effects of cerium oxide nanoparticles on the growth of keratinocytes, fibroblasts and vascular endothelial cells in cutaneous wound healing. Biomaterials.

[69] Rose S., Prevoteau A., Elzière P., Hourdet D., Marcellan A., Leibler L. (2014). Nanoparticle solutions as adhesives for gels and biological tissues. Nature.

[70] Meddahi-Pellé A., Legrand A., Marcellan A., Louedec L., Letourneur D., Leibler L. (2014). Organ Repair, Hemostasis, and In Vivo Bonding of Medical Devices by Aqueous Solutions of Nanoparticles. Angew. Chem. Int. Ed..

[71] Kim J.-H., Kim H., Choi Y., Lee D.S., Kim J., Yi G.-R. (2017). Colloidal Mesoporous Silica Nanoparticles as Strong Adhesives for Hydrogels and Biological Tissues. ACS Appl. Mater. Interfaces.

[72] Lozano-Fernández T., Dobrovolskaia M., Camacho T., González-Fernández Á., Simón-Vázquez R. (2019). Interference of Metal Oxide Nanoparticles with Coagulation Cascade and Interaction with Blood Components. Part. Part. Syst. Character.

[73] Barui A.K., Nethi S.K., Haque S., Basuthakur P., Patra C.R. (2019). Recent Development of Metal Nanoparticles for Angiogenesis Study and Their Therapeutic Applications. ACS Appl. Bio Mater..

[74] Kargozar S., Baino F., Hamzehlou S., Hamblin M.R., Mozafari M. (2020). Nanotechnology for angiogenesis: opportunities and challenges. Chem. Soc. Rev..

[75] Sanaeimehr Z., Javadi I., Namvar F. (2018). Antiangiogenic and antiapoptotic effects of green-synthesized zinc oxide nanoparticles using Sargassum muticum algae extraction. Cancer Nanotechnol..

[76] Kurtjak M., Aničić N., Vukomanovicć M., Kumavath R.N. (2017). Inorganic Nanoparticles: Innovative Tools for Antimicrobial Agents. Antibacterial Agents.

[77] Hench L.L., Jones J.R. (2015). Bioactive Glasses: Frontiers and Challenges. Front. Bioeng. Biotechnol..

[78] Hench L.L. (2006). The story of Bioglass. J. Mater. Sci. Mater. Med..

[79] Miguez-Pacheco V., Hench L.L., Boccaccini A.R. (2015). Bioactive glasses beyond bone and teeth: Emerging applications in contact with soft tissues. Acta Biomater..

[80] Pourshahrestani S., Kadri N.A., Zeimaran E., Towler M.R. (2019). Well-ordered mesoporous silica and bioactive glasses: promise for improved hemostasis. Biomater. Sci..

[81] Ostomel T.A., Shi Q., Tsung C.-K., Liang H., Stucky G.D. (2006). Spherical Bioactive Glass with Enhanced Rates of Hydroxyapatite Deposition and Hemostatic Activity. Small.

[82] Jebahi S., Oudadesse H., Jardak N., Khayat I., Keskes H., Khabir A., Rebai T., El Feki H., El Feki A. (2013). Biological therapy of strontium-substituted bioglass for soft tissue wound-healing: Responses to oxidative stress in ovariectomised rats. Ann. Pharm. Fr..

[83] Naseri S., Lepry W.C., Nazhat S.N. (2017). Bioactive glasses in wound healing: hope or hype?. J. Mater. Chem. B.

[84] Kargozar S., Mozafari M., Hamzehlou S., Baino F. (2019). Using Bioactive Glasses in the Management of Burns. Front. Bioeng. Biotechnol..

[85] Neščáková Z., Zheng K., Liverani L., Nawaz Q., Galusková D., Kaňková H., Michálek M., Galusek D., Boccaccini A.R. (2019). Multifunctional zinc ion doped sol—Gel derived mesoporous bioactive glass nanoparticles for biomedical applications. Bioact. Mater..

[86] Kantipudi S., Sunkara J.R., Rallabhandi M., Thonangi C.V., Cholla R.D., Kollu P., Parvathaneni M.K., Pammi S.V.N. (2018). Enhanced wound healing activity of Ag-ZnO composite NPs in Wistar Albino rats. IET Nanobiotechnol..

[87] Malka E., Perelshtein I., Lipovsky A., Shalom Y., Naparstek L., Perkas N., Patick T., Lubart R., Nitzan Y., Banin E. (2013). Eradication of Multi-Drug Resistant Bacteria by a Novel Zn-doped CuO Nanocomposite. Small.

[88] Oves M., Arshad M., Khan M.S., Ahmed A.S., Azam A., Ismail I.M.I. (2015). Anti-microbial activity of cobalt doped zinc oxide nanoparticles: Targeting water borne bacteria. J. Saudi. Chem. Soc..

[89] Rekha K., Nirmala M., Nair M.G., Anukaliani A. (2010). Structural, optical, photocatalytic and antibacterial activity of zinc oxide and manganese doped zinc oxide nanoparticles. Phys. B Condens. Matter.

[90] Guo B.-L., Han P., Guo L.-C., Cao Y.-Q., Li A.-D., Kong J.-Z., Zhai H.-F., Wu D. (2015). The Antibacterial Activity of Ta-doped ZnO Nanoparticles. Nanoscale Res. Lett..

[91] Nethi S.K., Rico-Oller B., Rodríguez-Diéguez A., Gómez-Ruiz S., Patra C.R. (2017). Design, synthesis and characterization of doped-titanium oxide nanomaterials with environmental and angiogenic applications. Sci. Total Environ..

[92] Dhanasekar M., Jenefer V., Nambiar R.B., Babu S.G., Selvam S.P., Neppolian B., Bhat S.V. (2018). Ambient light antimicrobial activity of reduced graphene oxide supported metal doped TiO_2_ nanoparticles and their PVA based polymer nanocomposite films. Mater. Res. Bull..

[93] Kim J., Kim H.Y., Song S.Y., Go S., Sohn H.S., Baik S., Soh M., Kim K., Kim D., Kim H.-C. (2019). Synergistic Oxygen Generation and Reactive Oxygen Species Scavenging by Manganese Ferrite/Ceria Co-decorated Nanoparticles for Rheumatoid Arthritis Treatment. ACS Nano.

[94] Wu H., Li F., Wang S., Lu J., Li J., Du Y., Sun X., Chen X., Gao J., Ling D. (2018). Ceria nanocrystals decorated mesoporous silica nanoparticle based ROS-scavenging tissue adhesive for highly efficient regenerative wound healing. Biomaterials.

[95] Jin C., Liu X., Tan L., Cui Z., Yang X., Zheng Y., Yeung K.W.K., Chu P.K., Wu S. (2018). Ag/AgBr-loaded mesoporous silica for rapid sterilization and promotion of wound healing. Biomater. Sci..

[96] Munusamy P., Sanghavi S., Varga T., Suntharampillai T. (2014). Silica supported ceria nanoparticles: A hybrid nanostructure to increase stability and surface reactivity of nano-crystalline ceria. RSC Adv..

[97] Shin K., Choi J.W., Ko G., Baik S., Kim D., Park O.K., Lee K., Cho H.R., Han S.I., Lee S.H. (2017). Multifunctional nanoparticles as a tissue adhesive and an injectable marker for image-guided procedures. Nature Commun..

[98] Wang X., Sun W., Yang W., Gao S., Sun C., Li Q. (2019). Mesoporous silica-protected silver nanoparticle disinfectant with controlled Ag^+^ ion release, efficient magnetic separation, and effective antibacterial activity. Nanoscale Adv..

[99] Zhu Y., Xu J., Wang Y., Chen C., Gu H., Chai Y., Wang Y. (2020). Silver nanoparticles-decorated and mesoporous silica coated single-walled carbon nanotubes with an enhanced antibacterial activity for killing drug-resistant bacteria. Nano Res..

[100] He L., Huang G., Liu H., Sang C., Liu X., Chen T. (2020). Highly bioactive zeolitic imidazolate framework-8-capped nanotherapeutics for efficient reversal of reperfusion-induced injury in ischemic stroke. Sci. Adv..

[101] Matter M.T., Starsich F., Galli M., Hilber M., Schlegel A.A., Bertazzo S., Pratsinis S.E., Herrmann I.K. (2017). Developing a tissue glue by engineering the adhesive and hemostatic properties of metal oxide nanoparticles. Nanoscale.

[102] Mädler L., Kammler H.K., Mueller R., Pratsinis S.E. (2002). Controlled synthesis of nanostructured particles by flame spray pyrolysis. J. Aerosol Sci..

[103] Matter M.T., Furer L.A., Starsich F.H.L., Fortunato G., Pratsinis S.E., Herrmann I.K. (2018). Engineering the Bioactivity of Flame-Made Ceria and Ceria/Bioglass Hybrid Nanoparticles. ACS Appl. Mater. Interfaces.

[104] Marie P.J., Ammann P., Boivin G., Rey C. (2001). Mechanisms of action and therapeutic potential of strontium in bone. Calcif. Tissue Int..

[105] Shahnazari M., Sharkey N.A., Fosmire G.J., Leach R.M. (2006). Effects of Strontium on Bone Strength, Density, Volume, and Microarchitecture in Laying Hens. J. Bone Miner. Res..

[106] Brandão-Neto J., Stefan V., Mendonça B.B., Bloise W., Castro A.V.B. (1995). The essential role of zinc in growth. Nutr. Res..

[107] Yamaguchi M. (1998). Role of zinc in bone formation and bone resorption. J. Trace Elem. Exp. Med..

[108] Lese I., Graf D.A., Tsai C., Taddeo A., Matter M.T., Constantinescu M.A., Herrmann I.K., Olariu R. (2018). Bioactive nanoparticle-based formulations increase survival area of perforator flaps in a rat model. PLoS ONE.

[109] Food and Drug Administration Guidance for Industry: Safety of Nanomaterials in Cosmetic Products. https://www.fda.gov/regulatory-information/search-fda-guidance-documents/guidance-industry-safety-nanomaterials-cosmetic-products.

[110] Fytianos G., Rahdar A., Kyzas G.Z. (2020). Nanomaterials in Cosmetics: Recent Updates. Nanomaterials.

[111] Albanese A., Tang P.S., Chan W.C.W. (2012). The Effect of Nanoparticle Size, Shape, and Surface Chemistry on Biological Systems. Ann. Rev. Biomed. Eng..

[112] Stark W.J. (2011). Nanoparticles in Biological Systems. Angew. Chem. Int. Ed..

[113] Home | Nanotechnology Characterization Lab (NCL). https://ncl.cancer.gov/.

[114] EUNCL | Nanomedicine Characterisation Laboratory. http://www.euncl.eu/.

[115] Yan L., Zhao F., Wang J., Zu Y., Gu Z., Zhao Y. (2019). A Safe-by-Design Strategy towards Safer Nanomaterials in Nanomedicines. Adv. Mater..

[116] Rösslein M., Liptrott N.J., Owen A., Boisseau P., Wick P., Herrmann I.K. (2017). Sound understanding of environmental, health and safety, clinical, and market aspects is imperative to clinical translation of nanomedicines. Nanotoxicology.

[117] Mârza S.M., Magyari K., Bogdan S., Moldovan M., Peştean C., Nagy A., Tăbăran F., Licarete E., Suarasan S., Dreanca A. (2019). Skin wound regeneration with bioactive glass-gold nanoparticles ointment. Biomed. Mater..

[118] Herrmann I.K., Beck-Schimmer B., Schumacher C.M., Gschwind S., Kaech A., Ziegler U., Clavien P.-A., Günther D., Stark W.J., Graf R. (2016). In vivo risk evaluation of carbon-coated iron carbide nanoparticles based on short- and long-term exposure scenarios. Nanomedicine.

[119] De Jong W.H., Borm P.J.A. (2008). Drug delivery and nanoparticles: Applications and hazards. Int. J. Nanomed..

[120] Liu R., Liu H.H., Ji Z., Chang C.H., Xia T., Nel A.E., Cohen Y. (2015). Evaluation of Toxicity Ranking for Metal Oxide Nanoparticles via an in Vitro Dosimetry Model. ACS Nano.

[121] Matusiewicz H. (2014). Potential release of in vivo trace metals from metallic medical implants in the human body: From ions to nanoparticles—A systematic analytical review. Acta Biomater..

[122] Sharifi S., Behzadi S., Laurent S., Laird Forrest M., Stroeve P., Mahmoudi M. (2012). Toxicity of nanomaterials. Chem. Soc. Rev..

[123] Soenen S.J., Rivera-Gil P., Montenegro J.-M., Parak W.J., De Smedt S.C., Braeckmans K. (2011). Cellular toxicity of inorganic nanoparticles: Common aspects and guidelines for improved nanotoxicity evaluation. Nano Today.

[124] Matter M.T., Li J.-H., Lese I., Schreiner C., Bernard L., Scholder O., Hubeli J., Keevend K., Tsolaki E., Bertero E. (2020). Multiscale Analysis of Metal Oxide Nanoparticles in Tissue: Insights into Biodistribution and Biotransformation. Adv. Sci..

